# DNACLUST: accurate and efficient clustering of phylogenetic marker genes

**DOI:** 10.1186/1471-2105-12-271

**Published:** 2011-06-30

**Authors:** Mohammadreza Ghodsi, Bo Liu, Mihai Pop

**Affiliations:** 1 Department of Computer Science, University of Maryland, College Park, MD 20742, USA; 2 Center for Bioinformatics and Computational Biology, University of Maryland, USA

## Abstract

**Background:**

Clustering is a fundamental operation in the analysis of biological sequence data. New DNA sequencing technologies have dramatically increased the rate at which we can generate data, resulting in datasets that cannot be efficiently analyzed by traditional clustering methods.

This is particularly true in the context of taxonomic profiling of microbial communities through direct sequencing of phylogenetic markers (e.g. 16S rRNA) - the domain that motivated the work described in this paper. Many analysis approaches rely on an initial clustering step aimed at identifying sequences that belong to the same operational taxonomic unit (OTU). When defining OTUs (which have no universally accepted definition), scientists must balance a trade-off between computational efficiency and biological accuracy, as accurately estimating an environment's phylogenetic composition requires computationally-intensive analyses. We propose that efficient and mathematically well defined clustering methods can benefit existing taxonomic profiling approaches in two ways: (i) the resulting clusters can be substituted for OTUs in certain applications; and (ii) the clustering effectively reduces the size of the data-sets that need to be analyzed by complex phylogenetic pipelines (e.g., only one sequence per cluster needs to be provided to downstream analyses).

**Results:**

To address the challenges outlined above, we developed DNACLUST, a fast clustering tool specifically designed for clustering highly-similar DNA sequences.

Given a set of sequences and a sequence similarity threshold, DNACLUST creates clusters whose radius is guaranteed not to exceed the specified threshold. Underlying DNACLUST is a greedy clustering strategy that owes its performance to novel sequence alignment and *k-*mer based filtering algorithms.

DNACLUST can also produce multiple sequence alignments for every cluster, allowing users to manually inspect clustering results, and enabling more detailed analyses of the clustered data.

**Conclusions:**

We compare DNACLUST to two popular clustering tools: CD-HIT and UCLUST. We show that DNACLUST is about an order of magnitude faster than CD-HIT and UCLUST (exact mode) and comparable in speed to UCLUST (approximate mode). The performance of DNACLUST improves as the similarity threshold is increased (tight clusters) making it well suited for rapidly removing duplicates and near-duplicates from a dataset, thereby reducing the size of the data being analyzed through more elaborate approaches.

## Background

Clustering of sequences (DNA or protein) is a common and basic analysis in bioinformatics that underlies many biological analyses. Clustering can be used to reveal underlying natural groupings of data. Clustering can also be used to simply reduce the size of a large dataset, such that a slower, more accurate, analysis can be applied [1]. The results of the slower analysis can then be carried over to the rest of the sequences. In this paper we focus on one application of DNA sequence clustering; namely the analysis of 16S ribosomal RNA (rRNA) data. The algorithms and principles underlying our tool should, however, be applicable to a wider range of sequence clustering tasks.

Sequence analysis of the 16S rRNA is one of the most commonly used methods for measuring microbial diversity and taxonomic composition of an environment. There are two complementary approaches to the analysis of 16S data: comparative classification, and unsupervised clustering. In the comparative approach, the taxonomic identity of a new sequence can be determined if it is similar to some of the sequences present in a curated database [2]. This approach, however, can not be reliably used for the analysis of novel sequences, thus scientists frequently rely on methods based on the unsupervised clustering of sequences [3-5]. Our work is specifically targeted at unsupervised methods. Note, however, that clustering of 16S sequences can be used as a pre-processing step even in the case of database-based methods in order to reduce the size of the datasets being analyzed and to speed up the classification process.

The traditional approach for clustering 16S rRNA sequences involves building a multiple sequence alignment (MSA) of all sequences, computing a pairwise distance matrix based on the MSA and clustering the resulting matrix [6]. The clustering algorithm is often a greedy hierarchical clustering algorithm which produces a rooted tree. The tree is then cut at some level, based on a specified similarity threshold, in order to construct a collection of clusters. Alternatively, if the taxonomic annotation of some of the sequences is known, the tree can be used in a more elaborate semi-supervised clustering algorithm [7].

Since the latest DNA sequencing technologies have become faster and cheaper, we are now faced with very large volumes of sequence data. Newer generations of sequencing technologies, e.g., 454 Life Sciences sequencing machines, can generate millions of sequences per run, each of which has a length of hundreds of base pairs. Such datasets cannot be easily clustered using the traditional approach outlined above.

First of all, finding the best multiple sequence alignment is computationally intractable - this problem falls into the category of NP-hard problems, i.e. problems that can only be solved by exploring an exponential number of possible solutions. Multiple sequence alignment tools rely on heuristic alignment algorithms that are not guaranteed to generate an optimal alignment (which is not a well defined concept, anyway).

The most common heuristic involves building a guide tree (a preliminary hierarchical clustering of the sequences) that then guides the construction of the multiple alignment. Often, the guide tree is constructed from a preliminary distance matrix constructed from pairwise alignments of the sequences - for large data this matrix is impractical (its size, and therefore time needed to construct it, grows with the square of the size of the datasets). Furthermore, determining a guide tree is difficult for large datasets since there could be many trees that fit the distance matrix equally well.

An alternative to the traditional clustering approaches that rely on multiple alignments, is a simple, yet effective, greedy clustering strategy. The process starts by selecting a sequence as a "seed" for a cluster. Additional sequences are added to this cluster if they fall within a certain distance from the seed. The process continues by selecting an unclustered sequence as the seed for a new cluster, and so on until all sequences have been clustered. This basic approach is employed by the programs CD-HIT [8],

UCLUST [9], and our own work. The main difference between these programs is in the way the clusters are constructed, specifically, how a program identifies all sequences that are nearby a cluster seed. As we will describe in more detail below, both CD-HIT and UCLUST search each sequence against a database of all previously constructed clusters. If the sequence does not have a good match against any of the existing clusters, it forms the seed for a new cluster.

The approach we present in this paper involves searching each cluster seed against a database of all unclustered sequences, thereby "recruiting" a set of sequences to the newly created cluster. We will show that this approach allows us to leverage an efficient search data structure to rapidly cluster large sets of sequences. Our approach is particularly well suited for high-stringency clustering (high similarity between the clustered sequences), intended to remove redundancy in the dataset by co-clustering sequences that are identical or whose differences are primarily due to sequencing errors. Representative sequences from each cluster can then be used as input to more computationally intensive analyses.

### Related Work

There are two popular tools designed for clustering large number of sequences: CD-HIT and UCLUST. CD-HIT [8] has been widely used in practice and is cited by hundreds of scientific articles. UCLUST [9] is a newer clustering tool. UCLUST is based on a fast sequence search algorithm (which is also used in the related USEARCH program), and can be more than an order of magnitude faster than CD-HIT. In the following we briefly review the algorithms used by these tools.

#### CD-HIT

CD-HIT uses a greedy incremental clustering algorithm. First the sequences are sorted in non-increasing order of their lengths. The first sequence becomes the first cluster representative. Each consecutive sequence is compared to all previously discovered cluster representatives, and is added to a cluster if it is within a user-selected distance threshold from the corresponding representative. Otherwise the sequence becomes the seed for a new cluster.

CD-HIT uses a "short word filtering" heuristic to avoid computing many of the costly pairwise alignments. Specifically, each sequence is represented as a *k*-mer spectrum (an array containing the number of occurrences of all substrings of length *k *in the sequence), and the initial comparison between sequences is performed between the corresponding spectra. If the *k*-mer counts differ significantly, it is unlikely that the sequences match each other well. CD-HIT relies on a statistical analysis to estimate the minimum number of *k*-mers that two sequences are expected to have in common, assuming they have a certain similarity to each other.

This filtering approach can be computationally expensive as it requires counting the number of *k*-mers shared by each sequence and all previously selected cluster representatives.

#### UCLUST

UCLUST follows virtually the same algorithm as CD-HIT, with two major exceptions: (i) sequences can be sorted in different ways, rather than simply by length (as done by CD-HIT); (ii) the mapping of sequences to existing cluster representatives is performed with a new search heuristic called USEARCH.

By default, UCLUST operates in an inexact mode. In the inexact mode each sequence is not aligned to all cluster centers found so far. Instead UCLUST sorts the cluster centers based on the number of "words" they have in common with the query. Each query sequence is thus aligned only with a few of the cluster representatives (up to a predefined constant), which are presumed most likely to be close to it. In the exact mode UCLUST operates more or less like CD-HIT, i.e. each query sequence is aligned to all cluster centers found so far. In this mode the word based filter is not used.

The inexact heuristic guarantees that the number of pairwise alignments is linear (and, thus, the algorithm is fast). In practice, this approximation could result in many more clusters than the exact mode, however the extent of this "blow-up" depends on the stringency of the clustering.

## Results

We describe the algorithms used by our tool in the following section. In the Testing section we evaluate the performance and quality of our implementation.

### Algorithm

The main goal in the design of our algorithms is computational efficiency and scalability. In this section we present a simple greedy clustering algorithm which avoids most of the pairwise comparisons in practice. The clustering algorithm uses an alignment search algorithm and a *k*-mer based filter which are also described in this section. Some definitions and general concepts are covered first.

#### Definitions

##### Distance measure

Clustering is intimately interconnected with the definition of the distance or similarity measure used to compare the objects being clustered. Several distance measures have been commonly used to compare sequence data, including *edit distance *(also called Levenshtein distance) - a measure that counts the minimum number of insertions, deletions, or substitutions that are required to transform a sequence into the other; *k*-mer *distance *- a measure of the number of substrings of length *k *that are shared (or differ) between two sequences; and *evolutionary distance *- an estimate of the number of evolutionary events (usually substitutions) that explain the differences between two sequences. In many cases more than one distance measure is used during clustering, e.g. a *k-*mer approach can be used to quickly discard sequences that should not belong to a same cluster, then a more precise, but slow, algorithm is applied (this combination is used by our algorithm as well as by CD-HIT and UCLUST).

Our approach defines the distance between two sequences to be the corresponding edit-distance where the cost function is simply unit cost for each gap or mismatch, and zero cost for matches. In the case of sequences of different lengths, we may want to allow gaps at the end and/or the beginning of the shorter sequence in the alignment, without penalty. This type of alignment is referred to as semi-global alignment. The default behavior of DNACLUST is to allow gaps at the end of a sequence but not at the beginning (aligned sequences are anchored at their 5' end), however other alignment policies can be selected through command-line parameters.

##### Clustering parameters

We define the *diameter *of a cluster as the maximum distance between any two sequences in the cluster. Our algorithm (like CD-HIT and UCLUST) returns one sequence per cluster as *cluster representative*. There is another related but slightly different concept of the *cluster center*, usually picked in such a manner that the maximum or average distance from it to the rest of the items in the cluster is minimized. In the following we sometimes loosely use the term cluster center to refer to the cluster representative. Given a cluster representative, the *cluster radius *can be defined as the maximum distance from the cluster representative to any sequence in that cluster. Given that the edit distance measure is a metric (follows triangle inequality) we also guarantee that the cluster diameter is at most twice the cluster radius.

##### Sequence similarity and sequence identity

The criterion used by UCLUST and CD-HIT to evaluate distance between sequences is the amount of sequence "identity", i.e. the fraction of characters that match exactly between the two sequences being aligned. This measure also defines the clustering stringency, e.g. a clustering threshold of 99% implies that the sequences within a cluster have 99% or higher identity. In an evolutionary sense identity is a natural definition of the similarity between two sequences, primarily because insertions and deletions are difficult to fit within evolutionary models - DNADIST [5] usually ignores any gaps when computing the distance matrix. The identity measure, however, can lead to unintuitive alignments, especially when comparing sequences of different lengths. In addition, identity is not a transitive measure, i.e. the fact that sequence A and B are identical (have zero distance according to the identity measure), and sequences B and C are identical, does not imply that sequences A and C are also identical. More generally, identity does not follow triangle inequality (A distance measure obeys triangle inequality if, for every three sequences *A*, *B*, and *C*, dist(*A*, *B*) + dist(*B*, *C*) ≤ dist(*A*, *C*)), implying that even though the cluster radius is small the cluster diameter may be high, i.e. while the distance between all the sequences in the cluster and the cluster representative is small, individual sequences may differ significantly from each other. As a result, the cluster is not as "tight" as would be implied by the distance threshold used. Furthermore, the resulting multiple alignment is of lower quality. (See Additional file 1, Figure S1.)

To avoid these problems we rely on a different measure of distance between sequences: the (semi-)global alignment score. In this context, the "similarity" between two sequences can be defined as:

Here the "length of the shorter sequence" refers to the length before alignment and does not include the gaps induced by the alignment.

Note, however, that due to the different definitions of distance, the clusterings produced by DNACLUST, CD-HIT, or UCLUST cannot be directly compared at a given clustering threshold.

##### Clustering properties

The clustering problem that we study in this work has the following form: given a set of sequences and a threshold on the cluster radius, group these sequences into clusters, and identify one sequence within each cluster as the cluster representative.

Given a metric distance between a set of items, any clustering of these items can have one or more of the following properties:

1. The radius of every cluster is less than or equal to the specified threshold.

2. The distance between any two cluster centers is strictly greater than the threshold.

3. The distance between any clustered item and any cluster center (except the center of the cluster to which the item belongs) is strictly greater than the threshold. This implies that the closest center to any item is the center of its cluster.

A clustering is *valid *if it satisfies property 1. A valid clustering with the minimum number of clusters is called an *optimal *clustering. Unfortunately, finding an optimal clustering (assuming a general metric distance between items,) is NP-hard [10]. A clustering that satisfies property 2 in addition to 1 is called an *exact *clustering. Our search and filter algorithms are designed to be able to create exact clusterings.

A clustering that satisfies property 3 is called a *well*-*separated *clustering. (For illustrations of exact and well-separated clusterings see Additional file 1, Figure S2.) Note that an exact clustering does not guarantee that the clusters are well separated. Also it is not always possible to cluster all of the items into well separated clusters. Below, we will describe an algorithm (that can be selected through command-line parameters) that provides well-separated clusterings.

#### Clustering Algorithm

The foundation of DNACLUST is a simple greedy clustering algorithm, which is similar to the algorithms used by CD-HIT and UCLUST.

In practice, the sequences are first sorted based on their length in a non-increasing order. Then at each iteration the longest remaining sequence is picked as the new cluster center. We form the largest possible cluster with this cluster center by searching through the set of unclustered sequences for all sequences that are less than a user-selected distance threshold from the cluster center. The clustered sequences are marked and are not taken into consideration any longer. (Pseudocode of this algorithm is provided in Additional file 1, Algorithm S1.)

Picking the longest unclustered sequence as the cluster center is necessary to ensure the correctness of clustering when the lengths of the sequences are not equal. Specifically if two sequences, both of which are longer than the cluster center, are clustered together, it is not possible to guarantee that they align well to each other, i.e. these sequences could be incorrectly placed in the same cluster even if they differ significantly.

In the case that all the sequences have (or are trimmed to have) the same length, it has been proposed that ordering the sequences by abundance results in a better clustering [11,12], especially in the presence of sequencing errors. The abundant sequences can be inferred to be the correct molecules that are surrounded by a "cloud" of imperfect sequences due to sequencing errors. This approach makes most sense if the data contain high coverage and relatively well separated sequences (e.g. data from a low-complexity community). In diverse communities, however, it can be difficult to distinguish between experimental noise and true genomic variation. In addition, determining "abundance" requires some form of clustering, either exact (counting the number of exact copies of each sequence in the data), or by allowing a small amount of error. DNACLUST is specifically targeted at such high stringency clustering applications, and, thus, could be used as an initial step in a more elaborate clustering scheme that takes abundance into account.

Note, however, that a full evaluation of the phylogenetic interpretation of clustering strategies is beyond the scope of our work. Our main goal was to develop an efficient and mathematically well-defined clustering approach. More complex analyses of the data that, e.g., take into account phylogenetic signal, can be performed by post-processing the output of our software. In order to generate an exact clustering, the search step must find all the unclustered sequences that are within the specified radius from the cluster center. In this context we refer to the cluster center as the *query sequence*. The distance measure can be based on either global alignment cost, or semi-global alignment cost, in which case the gap costs at one or both ends of the shorter sequence are ignored. Either of these policies can be picked by the user using the command line options. This search is the most time consuming step of the algorithm, and is described in more detail in the next section.

This algorithm can be easily modified to construct well separated clusters (property 3 in the previous section) as follows. Each time a new cluster of radius *r *is constructed, we also flag every unclustered sequence within distance 2*r *from the center of the new cluster. The flagged sequences can not be picked as cluster centers in subsequent iterations of the greedy algorithm, but may be included in a cluster constructed around an unflagged cluster center. This approach ensures that the distance between two cluster centers can not be less than two times the cluster radius. In order to implement this algorithm all we need to do is to double the search radius but only cluster the sequences that fall within the clustering threshold.

#### Alignment Search Algorithm

Our alignment search algorithm is designed to find all good (semi-) global alignments of the query sequence to a large set of sequences simultaneously. The main speed up is achieved by taking advantage of the fact that we are only interested in alignments that are high-quality: have a cost which is less than or equal to a certain threshold.

It is easier to explain this algorithm if we assume that all the sequences are stored in a trie data structure [13]. We traverse the trie using a depth first search (DFS) algorithm. At each internal node of the trie we compute the cost of best alignment of the query sequence (the representative for the current cluster) to the sequence corresponding to the path from root to the current internal node in the trie [14]. This corresponds to simultaneously matching the (identical) prefixes of all the sequences sorted in the trie within the subtree rooted at the current node with the query.

The pairwise alignment and the cost are computed using a dynamic programming algorithm, a variation of the Needleman-Wunsch algorithm [15].

First we describe the alignment algorithm for two complete sequences. Assume we are trying to align two sequences *S*_1 _and *S*_2 _of lengths *n*_1 _and *n*_2_. We fill an *n*_1 _**× ***n*_2 _table of numbers, such that the element at position (*i*, *j*) of the table - *T*_(*i*,*j*) _- contains the score (i.e. cost) of the "best" alignment of the first *i *characters of *S*_1 _to the first *j *characters of *S*_2_. In our case, the types of differences that are allowed are insertions, deletions and substitutions, and *T*_(*i*,*j*) _depends on only three other elements of the table:

If the table is filled row-by-row (or column-by-column), the total amount of computation needed to fill this table (and compute an alignment) is proportional to *n*_1 _**× ***n*_2_.

**Figure 1:**
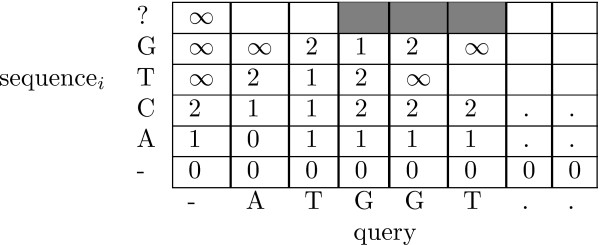
**Dynamic programming table example**. Partially filled dynamic programming table. The query sequence is represented on the horizontal axis. At this point the algorithm has computed the alignment costs for a prefix of length 4 of the data sequences - which is shown on the vertical axis. Since we are calculating semi-global alignment the first row is initialized to all zeroes, i.e. The alignment of the shorter data sequence can start at any position of the longer query sequence without any penalty. In this figure, the distance threshold is 2, and any values larger than this threshold are set to the maximum value represented by ∞. To optimize the running time, since there are only three valid values on the last finished row, only the values for the three gray cells need to be computed on row right above it.

The table is initialized as follows: If we are interested in a semi-global alignment (as in Figure 1) the first row is initialized to all zeros. On the other hand, if we assume all the sequences start at the same position (i.e. global alignment) the first row of the dynamic programming table is initialized with the cost of gaps required at the beginning of the alignment. The first column is always initialized with the cost of the required gaps.

Since we are trying to simultaneously align a query sequence to a set of sequences, we think of one of the sequences as growing (and shrinking) as we backtrack through the common prefixes of sequences in a suffix trie, and update the table as necessary. On the horizontal axis of the dynamic programming table (Figure 1) the query sequence is fixed. On the vertical axis we have the prefixes of the sequences in the trie. As we go deeper in the trie, the dynamic programming table is filled one row at a time. Each time the depth-first search of the trie traverses an edge, we only need to update one row of the table, namely the current top row. Also note that at each point the cost of the best semi-global alignment of the current path to the query is the minimum value on the top row.

Also, assuming unit cost for each gap (insertion or deletion), we do not even have to update all of the cells on the current row in the dynamic programming table after traversing an edge in the trie [16]. As is shown in Figure 1, the dynamic programming table updates need be performed only for the cells whose bottom-left neighbor contains a value no larger than the specified cluster radius.

This is due to the fact that for any two sequences *S*_1 _and *S*_2_, and any *i *and *j*, dist(*S*_1 _[1..*i*], *S*_2_[1..*j*]) **≥ **dist(*S*_1_[1..*i *- 1], *S*_2_[1..*j *- 1]) where dist() is the edit distance.

The main heuristic that speeds up this algorithm relies on the observation that it is not always necessary to compute the alignments of the query to all paths in the trie all the way to each leaf. Instead, during the depth-first search, if at any point the alignment cost of the prefix is too high, the recursive search terminates without further exploring the children of the current node in the trie.

The trie data structure described above is not explicitly built in our implementation. Instead we keep a list of the sequences *in lexicographically sorted order*. As we proceed to align one of the sequences (*S*) to the query we are also implicitly aligning all adjacent sequences that share a common prefix with *S*. In other words, each internal node of the virtual trie corresponds to a unique consecutive sub-list of the sorted list of sequences, in which the shared prefix of the sequences corresponds to the path from the root of the trie to that internal node.

Effectively we first try to align the first sequence to the query (in our case the cluster representative). If the alignment is good then the first sequence is added to the search results. When aligning the second sequence, and so on, we can avoid re-aligning its common prefix with the previously aligned sequence, thereby reducing the cost of computation. Note that if we fail to align the first say *α *characters of sequence *i *(*i*th sequence in the sorted list), and the common prefix of sequence *i *and sequence *i *+ 1 is longer than *α*, we do not need to try to align sequence *i *+ 1 at all. The search algorithm is very similar to the algorithm in [17], except that the backtracking threshold is fixed as the given radius of the clusters. We use the ternary quick sort algorithm [18] once, in the beginning, to sort the sequences lexicographically. The time spent for sorting the sequences is, however, much less than the time spent during clustering.

#### Star Multiple Sequence Alignment

One valuable output of a sequence clustering algorithm is a multiple sequence alignment representing the global relationship between the sequences present in the cluster. Such a multiple alignment can be used by users to manually inspect the quality of the clustering, and can also represent the substrate for more complex analyses (e.g. computation of evolutionary distances between the sequences).

We rely on a "star" multiple alignment heuristic that computes the multiple alignment from the pairwise alignments between each of the sequences and the cluster representative. The pairwise alignments between the sequences and the representative are a byproduct of our search algorithm. To construct the multiple alignment we reconcile the differences between these pairwise alignments by inserting gap characters as necessary. (For more detail see part 1 of Additional file 1.)

This approach guarantees that the pairwise distance between any two sequences in the alignment is at most twice the cluster radius (maximum distance between any sequence and the cluster representative).

#### Word-based Filter

Finding the best pairwise alignment of two sequences using dynamic programming is computationally intensive. It requires quadratic time in the length of the input sequences, in the general case. In our clustering application, however, it is possible to avoid calculating a pairwise alignment altogether, if we are certain that no good alignment exists. We use *k*-mer based filtering [8] to speed up the search for sequences with good alignment.

Given a sequence *S *of length *n *(e.g. one of the sequences we are trying to cluster), a *k*-mer is a substring of *S *of length *k*, where *k *is chosen to be much smaller than *n*. Sequence *S *contains *n *- *k *+ 1 overlapping *k*-mers, some of which may be identical.

The filter is based on the key intuition that if two sequences are within a small edit distance from each other, they must share most of their *k*-mers. In the following we formalize this idea.

Let us assign numbers from 1 to 4*^k ^*to the possible *k*-mers. Given a sequence *s*, by counting how many times each one of the 4*^k ^k*-mers appears in the sequence we obtain a vector of non-negative integers of dimension 4*^k ^*. Namely the *i*th element of this vector, *v_i _*, counts the number of times that *k*-mer number *i *appears in *s*. We call this vector the *k*-*mer spectrum *of the sequence *s*, and it is is denoted by spectrum*_k _*(*s*).

Given a vector of integer numbers, *v*, define pos(*v*) to be the sum of the positive values in the vector. Similarly define neg(*v*) to be the sum of the negative values. For example, for any sequence *s *of length *n*, we have pos(spectrum*_k _*(*s*)) = *n *- *k *+ 1 and neg(spectrum*_k _*(*s*)) = 0.

Consider two sequences, *s*_1 _and *s*_2_, that are close to each other. The following observation bounds the maximum difference between their *k*-mer spectra.

**Observation 1**. *If s*_2 _*has edit distance d from s*_1_, *then*, *for all k*, *pos*(*spectrum_k_*(*s*_1_) - *spectrum_k _*(*s*_2 _)) ≤ *k ***× ***d and neg*(*spectrum_k_*(*s*_1 _) - *spectrum_k _*(*s*_2_)) ≥ -*k ***× ***d*.

*Proof*. See part 2 of Additional file 1. □

Since we want to be able to report sequences that might have a good semi-global alignment to the query sequence, we have to consider the case in which one sequence is within a small edit distance from *a substring of *the other sequence. The following observation helps the handling of this case.

**Observation 2**. *If a sequence s*_1 _*is a substring of s*_2_, *then*, *for all k*, *neg*(*spectrum_k_*(*s*_2 _) - *spectrum_k _*(*s*_1_)) = 0.

**Lemma 1**. *If s*_1 _has edit distance d from s* and s* *is a substring of s*_2_, *then*, *for all k*, *pos *(*spectrum_k_*(*s*_1_) - *spectrum_k_*(*s*_2 _)) **≤ ***k ***× ***d*.

*Proof*. See part 2 of Additional file 1. □

Using Lemma 1, given a query sequence *q*, a sequence *s*, and a distance threshold *d*, if *for any k *we have

then we can be certain that no semi-global alignment of *s *to *q *exists which corresponds to a distance less than or equal to *d*.

Lemma 1 can be extended to quickly determine whether none of the sequences in a collection has a good alignment to the query sequence. We build a binary search tree of the sequences, and using their *k*-mer counts can quickly discard subtrees that do not have any sequence close to the query. (The algorithms for building and searching this binary tree are described in part 3 of Additional file 1.)

### Testing

In this section we compare clustering speed of DNACLUST with other clustering tools on different datasets. We also evaluate the quality of the multiple sequence alignment that DNACLUST can produce for each cluster.

#### Speed

DNACLUST and UCLUST can produce exact or approximate (i.e. inexact) clusterings. (The definitions of these terms are provided in the Algorithm section - *clustering properties*.) Creating an exact clustering takes more time. For these tools we have measured the running time in both settings.

We have created clusterings at different similarity/identity thresholds for each tool and each setting. (Sequence identity and sequence similarity are discussed in detail in the definitions part of the Algorithm section.) The radius of the clusters created range from 0.95 up to 0.995 similarity. (For a list of the parameters that can be set by the user of DNACLUST see part 4 of Additional file 1.)

**Figure 2:**
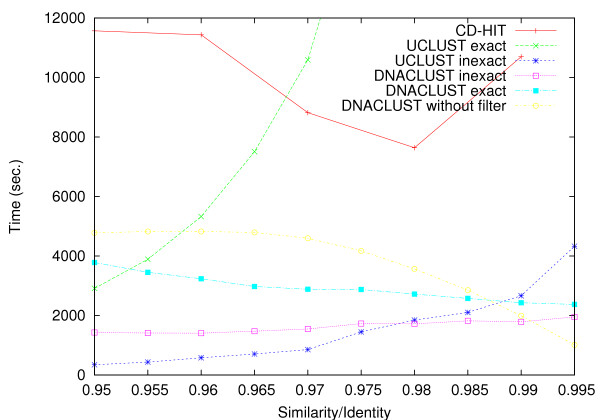
**Running times**. Plot of running time as a function of cluster radius for various tools and settings, on the twins dataset. The dataset contains 1.1 million pyrosequencing reads from the V2 region of the 16S rRNA gene. The reads have an average length of 231 base pairs. The running times were measured on a single 1.8 GHz processor of an Intel x86-64 Linux laptop with 4 GB RAM. The command line options were: dnaclust infile.fasta -s 0.9x -k 3 [--approximate-filter] > outfile.cluster uclust --input infile-sorted.fasta --uc outfile.cluster --id 0.9x [--exact].

In order to evaluate and compare the performance of our program, we use two publicly available datasets. The first dataset is from the gut microbiome of 154 individuals. These data were generated as part of a project to evaluate the differences in the gut microbiome of obese and lean twins [19]. The dataset contains 1.1 million pyrosequencing reads from the V2 region of the 16S rRNA gene. The reads have an average length of 231 base pairs. We refer to this dataset as the twins dataset. The running time of various clustering tools using different setting and cluster radii on the twins dataset is shown in Figure 2. The number of clusters generated for each setting is shown in Table 1.

**Table 1:** Number of clusters

	0.99	0.97	0.95
DNACLUST exact	233879	73726	28241

DNACLUST inexact	240125	76391	28661

UCLUST exact	144339	48418	20039

UCLUST inexact	253108	71361	26685

CD-HIT	245851	100280	55208

The number of clusters produced by DNACLUST, UCLUST and CD-HIT at various identity/similarity thresholds, on the twins dataset. Since each tool uses slightly different distance measures, the number of clusters can not be directly compared between different tools. (Namely the identity measure used by UCLUST and CD-HIT underestimates the distance between two sequences, as computed by the similarity measure used by DNACLUST). Instead we compare the change in the number of clusters when switching between the exact and inexact modes of each tool - a smaller change indicating better performance.

In exact mode the running time of UCLUST increases rapidly as the radius of the clusters is decreased. This is because a smaller cluster radius results in a large number of clusters (and hence cluster centers). In addition, for highly-similar sequences, the search heuristic used by UCLUST becomes less efficient. DNACLUST in exact mode is faster than UCLUST for any similarity threshold above 0.95.

UCLUST in inexact mode is much faster than in exact mode, and thus faster than DNACLUST in most cases. In inexact mode DNACLUST is faster than UCLUST only for similarity thresholds greater than or equal to 0.98. Both DNACLUST and UCLUST in inexact mode are roughly an order of magnitude faster than CD-HIT.

As seen in Table 1 the switch from exact to inexact mode leads to a significant change in the number of clusters generated by UCLUST, leading to up to 75% more clusters at the same similarity threshold. In other words, the improvement in speed comes at the cost of reduced cluster quality. In comparison, our *k*-mer filtering strategy (DNACLUST in inexact mode) leads to only a small increase in the number of clusters (**<**3%), i.e. our inexact heuristic is more effective in terms of speeding up the algorithm without significantly affecting the results of the clustering.

The second dataset contains all full 16S rRNA sequences from the Ribosomal Database Project [20]. We picked all of the sequences which were between 1300 and 1550 base pairs, covering almost the full length of the gene (480,312 sequences).

This test is meant to evaluate the performance of our algorithm on long sequences such as those that may be generated by future sequencing technologies. The running times of DNACLUST and UCLUST on the RDP dataset are shown in Table 2.

**Table 2:** Running times on RDP dataset

	0.99	0.97	0.95
DNACLUST exact	204	372	960

UCLUST exact		7800	5040

DNACLUST inexact	74	76	150

UCLUST inexact	43	29	16

The running times (minutes) of DNACLUST and UCLUST with various similarity thresholds on the RDP dataset.

The running times were measured on a single 2.8GHz processor of an AMD64 Linux workstation. The command line options were:

dnaclust infile.fasta -l -s 0.9x -k 5 [--approximate-filter] > outfile.cluster uclust --input infile-sorted.fasta --uc outfile.cluster --id 0.9x [--exact]

The sequences in the RDP dataset are almost three times longer than the earlier dataset, allowing us to evaluate how our approach scales with anticipated increases in read length. The alignment algorithm is slower on these data since, at the same level of similarity/identity between sequences, the total number of differences is higher. The trends in the performance are, however, consistent with the results observed on the twins dataset. DNACLUST still outperforms UCLUST for tighter clustering thresholds (**>**0.95): UCLUST in the exact mode takes more than 130 hours to cluster this dataset, in contrast to just over 6 hours for DNACLUST.

Please note that the results shown above ignore any connection between clusters and actual biological entities, i.e. we are primarily concerned with whether the clustering is mathematically consistent instead of whether clustering captures some underlying biological truth. In general, no fixed clustering threshold adequately captures the taxonomic structure in the data [6], in part because "biological truth" is not a well defined concept (at least not in mathematical terms). Additionally, sequencing errors can blow up the number of clusters especially if the sequencing error rate is of roughly the same order of magnitude as the clustering threshold.

To estimate the true taxonomic composition of a dataset we recommend a two step process that starts by building tight clusters (e.g. at 0.99 similarity) with DNACLUST, then uses the cluster representatives (and the size of the clusters) as input for a more sophisticated but slower algorithm which could not otherwise be applied to the original dataset.

#### Multiple Sequence Alignment

In the following we compare the quality of the multiple sequence alignment (MSA) produced by DNACLUST and commonly-used multiple alignment algorithms. We are defining the quality of a multiple sequence alignment in a very strict sense, specific to the analysis of 16S rRNA data: we measure how well the distance matrix computed by DNADIST (a commonly used tool that estimates evolutionary distances between sequences in a multiple alignment) matches the clustering criteria: i.e. if the clustering threshold (i.e. radius) is 99% identity, we expect the largest value in the distance matrix (i.e. largest diameter) to be lower than 2%.

To evaluate the quality of the multiple sequence alignment produced by our program we compare it to two of the most popular MSA tools, ClustalW [21] and MUSCLE [22]. Neither of these tools can handle as many sequences as our largest clusters contain (the largest cluster of the twins dataset at 95% similarity contains 74,465 sequences). Both of these tools crash when they fail to allocate enough memory for an *n ***× ***n *matrix to store the pairwise distances, where *n *denotes the number of input sequences.

For *n *≥ 38, 000 ClustalW, and for *n *≥ 22, 000 MUSCLE could not run on a machine with 4 GB of RAM. Note that larger datasets could be aligned using MUSCLE given additional RAM or by specifying the main memory limit (-maxmb parameter). The total memory requirements, however, grow quadratically as a function of the number of sequences being aligned, ultimately limiting the size of datasets that can be analyzed on commodity hardware.

To compare the multiple alignment routines, we selected one of the clusters produced by DNACLUST within the twins dataset. This cluster was constructed with a 95% similarity threshold (5% cluster radius), and contained 1117 sequences. These sequences were aligned using MUSCLE and ClustalW (with and without the "-quicktree" option), resulting in three multiple sequence alignments. We compared these "traditional" MSAs to those generated by DNACLUST and UCLUST. A pairwise distance matrix was obtained based on each MSA using DNADIST [5]. The maximum distance between any pair of the sequences was then reported, which corresponds to the diameter of the cluster in terms of evolutionary distances inferred from the corresponding MSA. The running time and the inferred cluster diameter for different MSA tools are shown in Table 3.

**Table 3:** Multiple sequence alignment building times

MSA method	Time (sec.)	Diameter (DNADIST)
ClustalW	1545.5	0.251

ClustalW -quicktree	87.6	0.264

MUSCLE	197.8	0.198

UCLUST	0.1	0.156

DNACLUST	0.8	0.094

Time spent building a Multiple Sequence Alignment of a sample cluster using different tools, and the diameter of the MSA produced, as reported by DNADIST. The diameter is expected to be less that or equal to 0.10.

This experiment validates the clusters and corresponding MSAs produced by DNACLUST using an independent tool for measuring evolutionary distance between DNA sequences. The DNADIST distance matrices are the underlying data used in the "traditional" 16S rRNA clustering approaches. The results also demonstrate that commonly used multiple sequence alignment tools are not well suited for the alignment of a large number of sequences.

DNACLUST and UCLUST rely on a star-alignment heuristic, and use the cluster representative as the one sequence against which all the other sequences are aligned. This approach guarantees that the distance between any pair of sequences within the MSA is at most twice the radius of the cluster. Furthermore, this approach is efficient: building a star MSA only requires time linear in the number of sequences, in contrast to the quadratic time needed to construct the guide tree in most traditional multiple alignment approaches. We further compared multiple sequence alignments produced by DNACLUST and UCLUST. We built clusterings using both programs (and multiple sequence alignments for each cluster) from the twins dataset at the thresholds 95%, 97% and 99% (6 clusterings in total). Since computing pairwise distances is computationally intensive especially for large clusters, from each clustering we randomly selected 50 clusters containing between 100 and 500 sequences. For each cluster we also built a multiple alignment using ClustalW.

**Figure 3:**
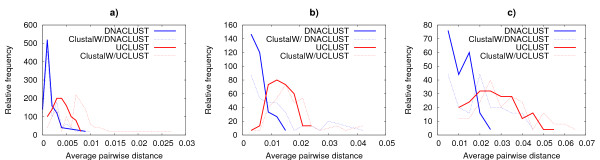
**Distribution of cluster MSAs based on their average pairwise distance**. Figures 3a, 3b and 3c show the distribution of sampled cluster multiple sequence alignments based on their average pairwise distance for thresholds 99%, 97% and 95%, respectively. The figures show that DNACLUST cluster MSAs (thick blue line) are tighter (i.e. have smaller average pairwise distance) than UCLUST cluster MSAs (thick red lines). Furthermore computing a "traditional" MSA using ClustalW from the clusters produced by DNACLUST and UCLUST results in an overestimation of the distances between sequences (dashed lines).

We generated the pairwise distance matrix for each alignment using DNADIST, then computed the average pairwise distance of the aligned sequences. Figure 3 show the distribution (relative frequency) of the alignments based on their average pairwise distance for each threshold. The clusters produced by DNACLUST are tighter than the clusters produced by UCLUST at the same threshold because of the more stringent definition of distance between sequences (we rely on full alignment score while, by default, UCLUST uses identity). Closer examination of the multiple sequence alignments produced by UCLUST shows that they tend to contain more gaps (which is consistent with the UCLUST definition of distance as sequence identity). Average frequencies of gaps in multiple sequence alignments produced by DNACLUST and UCLUST at various thresholds are shown in Table 4. Note that the gaps do not affect the pairwise distance computation, as they are not penalized by DNADIST.

**Table 4:** Average frequency of gaps in the multiple sequence alignments

	0.99	0.97	0.95
DNACLUST	0.016	0.071	0.103

UCLUST	0.071	0.117	0.146

The average frequency of gaps in multiple sequence alignments for sampled clusters at various similarity thresholds. For each MSA, the frequency of gaps is the number of gaps divided by the total number of characters in the MSA. Gaps before the beginning and after the end of each sequence are excluded. Note that since an insertion in one sequence results in a gap in all other sequences in the MSA, the ratio of gaps may be higher than the clustering threshold. Since the sequence identity measure used by UCLUST does not take gaps into account the number of gaps in UCLUST MSAs are higher than the gaps in DNACLUST MSAs, specially at more stringent thresholds.

Our results also show that the traditional approach for computing multiple alignments (here using ClustalW) results in an overestimate of the distances between the aligned sequences (dashed lines in Figure 3), confirming the observation that the star alignment strategy is more appropriate for large sets of highly similar sequences.

## Availability and requirements

**Project name: **DNACLUST

**Project home page: **http://dnaclust.sourceforge.net/

**Operating system: **Linux - x86/x86-64

**Programming language: **C++

**Other requirements: **Boost C++ libraries 1.34 or higher

**License: **GNU GPL

## Discussion

Clustering is a basic problem in computational biology and other sciences. It is, however, a difficult problem. Finding the optimal solution, even in very simple models, is computationally prohibitive. Considering the fact that the amount of data created by sequencing machines is growing at a rapid pace (outpacing, in fact, Moore's law at the moment), efficient algorithms for clustering are needed.

In this paper we have focused on a simple greedy clustering approach. Our algorithm's running time depends on the data and the cluster radius threshold, and is particularly effective at high clustering thresholds. The efficiency of our algorithm is due to a new *k*-mer filtering approach, and to an efficient search strategy that allows us to quickly recruit sequences that could be assigned to a cluster. The software implementation is open-source and competitive with other commonly used clustering tools.

It should be noted that our focus has been simplicity, generality and performance. We do not claim to discover the underlying biological "truth". Such analysis require more sophisticated algorithms and are much more computationally intensive. Our tool can help in reducing the size of the data so that these, more intensive, analyses can be applied. What we provide is a mathematically well defined clustering, which is the best we can hope for given that the biological truth has yet to be unambiguously characterized in mathematical terms.

We have compared our tool to state of the art software for clustering large numbers of sequences. An interesting observation is that the running time of DNACLUST decreases as the radius of the cluster is decreased, whereas the running time of UCLUST increases. Our search algorithm is more effective at higher stringencies, while the heuristics used by UCLUST are more effective when sequences are more dissimilar. This suggests that a faster algorithm could be developed that combines the best properties of both approaches.

Our *k*-mer based filter and alignment search algorithms can also be used for searching against databases that contain a large number of short sequences. In this scenario, the fact that our filter is completely sensitive is very useful. If the sequence database is static, our search data structures can be built once and stored on the disk for subsequent queries. We hope to add this functionality in future versions.

Finally, it is important to point out that there is still the need for even faster clustering tools. For very large datasets (over several million sequences), such as the ones produced by Human Microbiome Project, all available tools take more than a few days to run. Higher performance can possibly be achieved through parallel computation, and we intend to explore such approaches in future versions of our software.

## Conclusions

The datasets analyzed by biologists are rapidly increasing in size and efficient clustering algorithms are necessary to help reduce the effective size of these datasets. Here we presented a novel approach for sequence clustering that is particularly well suited for high-stringency clustering, outperforming other state of the art clustering tools in this context. While our focus has been the analysis of 16S rRNA sequences, the algorithms we describe can be applied in other contexts as well, e.g. to identify duplicates in high-throughput sequencing data.

While more relaxed clustering thresholds are often used in metagenomic studies (≤ 97% similarity), using any fixed threshold is not a good approach for creating biologically meaningful clusters [6]. Here we have focused on creating rigorously defined, tight clusters. The representatives of these clusters can be used in further analysis (e.g. to create phylogenetically-informative clusters using more computationally intensive methods), in effect reducing the size of the original dataset.

The software implementing our clustering approach is freely available under an open-source license.

## Competing interests

The authors declare that they have no competing interests.

## Authors' contributions

MG and MP designed the algorithms. MG developed the program. BL evaluated UCLUST. MG and MP wrote the manuscript. All authors have read and approved the manuscript.

## Supplementary Material

Additional file 1 **Additional information**. Proofs of the lemmas, details of the *k*-mer filter algorithm, pseudocode, illustrations and description of the program arguments are provided in the additional information. Click here for file
