# Supplementary material for: DNACLUST: accurate and efficient clustering of phylogenetic marker genes
Source: BMC Bioinformatics. 2011 Jun 30;12:271. doi: 10.1186/1471-2105-12-271 (PMC3213679; doi:10.1186/1471-2105-12-271)
Supplement: Additional file 1 — Additional information. Proofs of the lemmas, details of the k-mer filter algorithm, pseudocode, illustrations and description of the program arguments are provided in the additional information. [file 1471-2105-12-271-S1.PDF]

Additional information

Figure S1: The multiple alignment of a cluster produced by UCLUST. Note that the sequences match at 100% *identity*, despite the long gaps.

```
>0|100.0%|nomatch
--GATCTCTCCGGA----------------------------GATCTCGTAGCGCTCATC------------------------------CACTCCTTCACCGGGCTCTGCCCCTTT---
>0|100.0%|dnaGfrag
--------TCCGGAGACGATCGCGCTCGTGAAAGAGCGCACGGATCTCGTAGCGCTCA--------------------------------------------------------------
>0|*|dnaG
ATGATCTCTCCGGAGACGATCGCGCTCGTGAAAGAGCGCACGGATCTCGTAGCGCTCATCGGCGAAAGCGTGCGCCTCGCCCGCCGAGGGCACTCCTTCACCGGGCTCTGCCCCTTTCAC
```

Figure S2: Two possible clusterings of a set of points on the plane based on Euclidean distance. The cluster centers are colored in black. In both of these clusterings no two cluster centers are closer than the cluster radius from each other. The well separated clustering guarantees that each point is assigned to nearest cluster center, a guarantee not provided by an exact clustering. Note, however, that while an exact clustering can cluster every point in the data-set, this may not be possible in the well separated clustering (gray points in the figure).

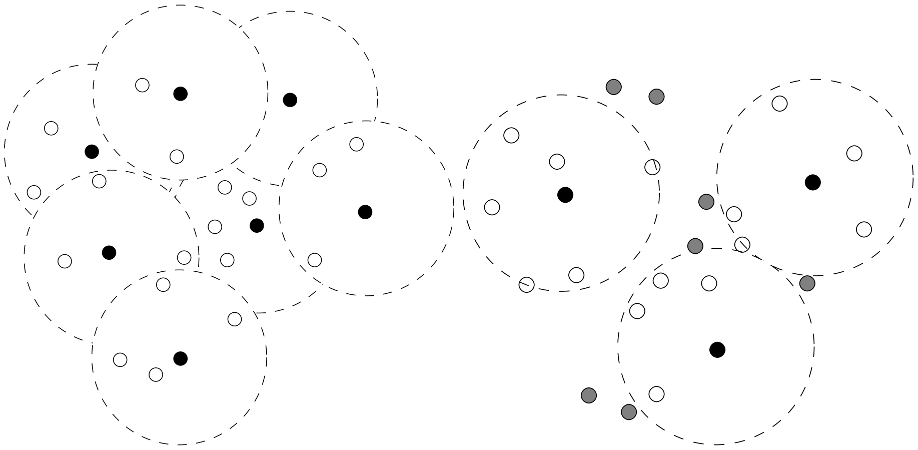

(a) Exact clustering (b) Well separated clustering

1. Building a star multiple sequence alignment from pairwise alignments to the cluster center: Consider two consecutive letters of the cluster center sequence; in each pairwise alignment, there could be different numbers of gaps between these two letters. In the multiple alignment we need the number of gaps between these two letters to be the maximum of the numbers of gaps inserted between these characters in any of the pairwise alignments.

2. Proof of the word-based filter lemmas:

Algorithm S1 Greedy Clustering Algorithm

$i \leftarrow 1$
while Sequences $\neq \emptyset$ **do**
  $\text{Center}_i \leftarrow \text{longest}(\text{Sequences})$
  $\text{Cluster}_i \leftarrow \text{search}(\text{Sequences}, \text{Center}_i, \text{RADIUS})$
  $\text{Sequences} \leftarrow \text{Sequences} - \text{Cluster}_i$
  $i \leftarrow i + 1$
end while

Observation 1. *If s_2 has edit distance d from s_1, then, for all k,* $pos(spectrum_k(s_1) - spectrum_k(s_2)) \leq k \times d$ *and* $neg(spectrum_k(s_1) - spectrum_k(s_2)) \geq -k \times d$.

Proof. Assume $d = 1$. Then s_2 can be obtained from s_1 by one insertion or one deletion or one substitution. Consider $\text{spectrum}_k(s_1)$. It is easy to check that in all three cases, at most k new k-mers are created, also at most k existing k-mers are eliminated.

If $d > 1$, s_1 can be transformed into s_2 by applying d edits one after the other. During this process a set of $d - 1$ intermediate sequences are obtained. Note that the edit distance between each consecutive pair of the intermediate sequences is exactly 1. Therefore the total number of new k-mers in s_2 is at most $k \times d$. Similarly, the total number of k-mers eliminated from s_1 is also at most $k \times d$. □

Lemma 1. *If s_1 has edit distance d from s^* and s^* is a substring of s_2, then, for all k,* $pos\,(spectrum_k(s_1) - spectrum_k(s_2)) \leq k \times d$.

Proof. By Observation 1 and Observation 2 and algebra:

$$
\begin{aligned}
&\text{pos}\,(\text{spectrum}_k(s_1) - \text{spectrum}_k(s_2)) \\
&= -\,(\text{neg}\,(\text{spectrum}_k(s_2) - \text{spectrum}_k(s_1))) \\
&\leq -\,(\text{neg}\,(\text{spectrum}_k(s_2) - \text{spectrum}_k(s^*)) + \text{neg}\,(\text{spectrum}_k(s^*) - \text{spectrum}_k(s_1))) \\
&\leq -(0 + (-k \times d)) \\
&= k \times d
\end{aligned}
$$

□

Using Lemma 1, given a query sequence q, a sequence s, and a distance threshold d, if *for any* k we have

$$\text{pos}\,(\text{spectrum}_k(s) - \text{spectrum}_k(q)) > k \times d$$

then we can be certain that no semi-global alignment of s to q exists which corresponds to a distance less than or equal to d.

3. Extension of Lemma 1 to quickly determine if none of the sequences in a collection has a good alignment to the query sequence: Given a set of n vectors which have the same dimension define $\min_1(v_1, v_2, \ldots, v_n) = v$ to be the vector of equal dimension, such that $v[i] = \min(v_1[i], v_2[i], \ldots, v_n[i])$, for all i. Given a set of n sequences, define

$$\text{min\_spectrum}_k(s_1, \ldots, s_n) = \\ \min_1(\text{spectrum}_k(s_1), \ldots, \text{spectrum}_k(s_n))$$

Similarly we define $\max_1()$ and $\text{max\_spectrum}_k()$. The following corollary follows from Lemma 1.

Corollary 1. *Given a query sequence q and a set $S = \{s_1, \ldots, s_n\}$ of sequences, for any k, if*

$$pos(min\_spectrum_k(s_1, \ldots, s_n) - spectrum_k(q)) > k \times d$$

Then none of the sequences in S is within edit distance less than or equal to d from q.

We use this result to quickly discard sequences that could not possibly be close to a given query sequence. The maximum length of k-mers used is denoted by $k_{\max}$. The value of $k_{\max}$ is a parameter that can be specified by the user. At the beginning of the program, a search data structure is built based on the $k_{\max}$-mer spectra of the input sequences. The data structure we use for the filter is a binary tree, in which the (unique spectra of the) input sequences are the leaves. The root of the tree represents the set of all of the sequences. For each internal node let descendants(node) denote all the leaf sequences that are located under this node. At each internal node of this tree the following information is stored: for every $k = 1 \ldots k_{\max}$ we store two vectors: min_spectrum_k(descendants(node)) and max_spectrum_k(descendants(node)).

Let us first explain how we search for the sequences potentially close to a query sequence based on their k-mer spectrums, if such a binary tree is already constructed. We first calculate the k-mer spectrum of the query for every $k = 1, \ldots, k_{\max}$. We use a recursive search function shown in Algorithm S. The parameters for the first call of this function are the root node of the search tree and $k = 1$. If at some internal node $\text{pos}(\text{node.min\_spectrum}_k - \text{query\_spectrum}_k) > k \times d$ then by Corollary 1, none of the descendants of this node can be close enough to query. If $\text{pos}(\text{node.max\_spectrum}_k - \text{query\_spectrum}_k) \leq k \times d$, then every descendant is acceptable regarding this k value. If $k = k_{\max}$ we return all descendants, otherwise we increment k and continue the search. Finally if none of the cases above happens, we recursively search the two children of the current node, and return the union of results from each one.

Let us now explain how the binary tree is built, to allow the search algorithm to quickly find potentially close sequences. Intuitively the sequences with similar spectra should be grouped together. In case of k-mer spectra we measure similarity by the L_1 distance. We build the tree using a top-down recursive

Algorithm S2 k-mer Filter Search Algorithm

```
function search(Node node, Integer k)

if pos(node.min_spectrum_k − query_spectrum_k) > k × d then
  return ∅
end if
if pos(node.max_spectrum_k − query_spectrum_k) ≤ k × d then
  if k < k_max then
    return search(node , k + 1)
  else
    return descendants(node)
  end if
end if
return search(node.left_child, k) ∪ search(node.right_child, k)
```

procedure, based on $k_{\max}$-mer spectra. At each step if the number of unique k-mer spectra is greater than one, we divide them into two groups and recursively build a binary search tree of each group. The set of k-mer spectra is divided into two using the 2-means clustering algorithm. This is a special case of the k-means (Note that the variable k in k-means algorithm denotes the number of clusters, and is unrelated to the variable which denotes the k-mer length.) algorithm with $k = 2$. In our implementation of k-means in L_1 distance we define the centroid of a cluster to be the median of the values in each dimension instead of their mean (which is the more commonly used definition).

As the greedy clustering of Algorithm S progresses the number of sequences not yet clustered decreases. In practice the clusters created early in the algorithm are bigger, and most of the clusters created near the end of the algorithm are singletons. Therefore, to speed up the filter, the binary search tree structure is rebuilt, when the number of remaining sequences shrinks sufficiently. Specifically, we rebuild our tree data structure every time the number of sequences not clustered so far drops to less than half of the sequences in the structure.

4. Running DNACLUST: In its simplest invocation, DNACLUST expects only three parameters: (i) a multi-FASTA file containing the input sequences; (ii) a similarity threshold; and (iii) the name of the output file to contain the resulting clustering. The output file is a simple text file containing one line per cluster. Each line starts with the identifier of the cluster representative, followed by a space-delimited list of the identifiers of all the sequences in the cluster. This file format is augmented with the FASTA records corresponding to the aligned sequences, if the multiple sequence alignment option is selected.

DNACLUST can be configured through several command line options, briefly described below.

1. *similarity:* Similarity threshold specifies the radius of the clusters created.

The threshold is a numerical value between 0 and 1.

2. *k-mer length:* The *maximum* length of k-mers used for filtering. The longer k-mer lengths require more memory to store k-mer counts and the filtering will be slower. However with the longer k-mer length, the filter will be more specific and therefore the sequence alignment search will be faster. If the median length of the input sequences is l then a good choice (and the default value) for k-mer length is $\log_4(l)$.

3. *multiple alignment:* Produces a multiple sequence alignment for each cluster.

4. *allow left gaps:* By default the sequences are anchored at the 5' end. This option allows for the cluster sequence to be aligned to any substring of the cluster center sequence. i.e. The gaps at both ends are not penalized when computing the alignment score.

5. *approximate filter:* Use heuristics to speed up the k-mer filter algorithm. The downside is that the clustering that is produced will not necessarily be an exact clustering. This may slightly increase the number of clusters created.

6. *well separated clusters:* Use a heuristic clustering strategy that guarantees the clusters to be well separated, i.e. each clustered sequences is guaranteed to be closer to the corresponding cluster center than to the center of any other cluster.
